# Experiences of a Digital Behavior Change Intervention to Prevent Weight Gain and Promote Risk-Reducing Health Behaviors for Women Aged 18 to 35 Years at Increased Risk of Breast Cancer: Qualitative Interview Study

**DOI:** 10.2196/57964

**Published:** 2024-11-25

**Authors:** Rhiannon E Hawkes, Mary Pegington, Alan Davies, Julia Mueller, Anthony Howell, D Gareth Evans, Sacha J Howell, David P French, Michelle Harvie

**Affiliations:** 1 Manchester Centre for Health Psychology School of Health Sciences University of Manchester Manchester United Kingdom; 2 Division of Cancer Sciences University of Manchester Manchester United Kingdom; 3 The Prevent Breast Cancer Research Unit The Nightingale Centre Manchester University NHS Foundation Trust Manchester United Kingdom; 4 Division of Informatics Imaging and Data Sciences University of Manchester Manchester United Kingdom; 5 Medical Research Council Epidemiology Unit University of Cambridge Cambridge United Kingdom; 6 Manchester Breast Centre Oglesby Cancer Research Centre, The Christie NHS Foundation Trust University of Manchester Manchester United Kingdom; 7 Genomic Medicine, Division of Evolution, Infection and Genomic Sciences University of Manchester St Mary’s Hospital, Manchester University NHS Foundation Trust Manchester United Kingdom; 8 Department of Medical Oncology The Christie NHS Foundation Trust Manchester United Kingdom

**Keywords:** breast cancer, health behavior, weight gain, weight control, BMI, app, acceptability, feasibility

## Abstract

**Background:**

Breast cancer is the most common form of cancer in women. Adult weight gain and modifiable health behaviors, including smoking, alcohol intake, and lack of physical activity, are well-known risk factors. Most weight gain in women occurs between the ages of 18 and 35 years. Digital interventions have the potential to address logistical challenges that arise in reaching women in this age range. We designed a digital intervention targeting weight gain prevention and other modifiable health behaviors for young women at increased risk of breast cancer. Women aged 18 to 35 years were recruited to this single-arm intervention study over 2 months to test the acceptability and usability of the intervention, which comprised a group welcome event held via videoconferencing, app, and private Facebook group.

**Objective:**

This nested qualitative substudy explored women’s views and experiences of being part of the digital health intervention to inform future intervention development for a feasibility study.

**Methods:**

A total of 20 women aged 23 to 35 years who were at increased risk of breast cancer were interviewed via telephone within 1 month after completing the intervention, between February 2023 and March 2023. The women were asked about their experiences of the digital intervention and the extent to which it may have influenced their health behaviors. Data were analyzed thematically and organized using the framework approach.

**Results:**

The interviews lasted for a median of 37 (IQR 30-46) minutes. Overall, the women perceived the digital health intervention comprising education, tracking, and support to be acceptable for weight gain prevention. In total, 4 themes were generated. *A “missed opportunity” in breast cancer prevention services* encompasses the lack of services that currently exist for young women at increased risk of breast cancer. *The pros and cons of being part of a community* encompasses the divergent views that the women had regarding engaging with other women at increased risk. *The importance of an interactive app* focuses on features that the women would want from the app to promote engagement with the intervention. *The different wants and needs of different age groups* highlights that an intervention such as this one would need to be customizable to suit the needs of women at different life stages.

**Conclusions:**

There is an unmet need in prevention services for young women aged 18 to 35 years at increased risk of breast cancer. The women perceived the app to be an acceptable intervention for weight gain prevention but emphasized that the intervention would need to be customizable to meet the needs of different age groups within the group of women aged 18 to 35 years. The digital intervention could be a scalable behavior change strategy for UK family history clinics.

## Introduction

### Background

Breast cancer is the most common form of cancer in women, with >55,000 diagnoses in the United Kingdom each year, and figures are predicted to increase [1]. In the United Kingdom, women aged 50 to 70 years are invited by the National Health Service breast screening program for 3-yearly mammograms. Guidance from the UK National Institute for Health and Care Excellence recommends that women who are known to be at increased risk of breast cancer (due to a strong family history of breast or ovarian cancer) should be offered annual breast cancer screening from the age of 40 years [2]. In the United Kingdom, women at increased risk of breast cancer have the option to attend family history clinics, which play a potentially important role in breast cancer prevention as women at increased risk experience proportionally more cancers. Access is currently via self-referral of concerned women or via general practitioner referral. Family history clinics undertake comprehensive assessments of breast cancer risk using established risk assessment tools (eg, the Tyrer-Cuzick model [3] or the CanRisk tool [4]). Those found to have an estimated lifetime risk are entered into these clinics for breast cancer surveillance and the offer of risk-reducing interventions (eg, surgery and risk-reducing medications such as tamoxifen [2]).

National Institute for Health and Care Excellence guidance recommends that family history clinics provide advice on healthy weight and health behaviors (ie, limited alcohol consumption, avoidance of smoking, and physical activity) to lower breast cancer risk [2]. These factors have an equal or greater effect on relative risk of breast cancer among women who have a family history of breast cancer compared to those without a family history [5-8]. Many women attending these clinics have obesity or overweight (60%) and health behaviors associated with risk (ie, 30% fail to meet physical activity recommendations, and 45% exceed alcohol consumption recommendations [9]). Hence, there is an unmet need to implement prevention through weight control and health behavior change (ie, reduced alcohol consumption, smoking cessation, and increased physical activity) in these clinics.

We previously highlighted that most weight gain in women occurs between the ages of 18 and 35 years [9], and once weight is gained, it is often difficult to lose [10]. Therefore, preventing weight gain in young women will potentially be more effective than dealing with weight problems once they have occurred. Records from the Manchester Family History Clinic since 1987 show that 23.5% of women are seen by the family history clinic and provided with their estimated breast cancer risk before the age of 35 years (Prof Gareth Evans, personal communication). Women are then asked to return to the clinic some years later when they can start breast cancer screening and can be offered preventive medicines. These women do not receive any further behavior change support in the interim. Digital interventions (eg, apps, social media, and wearable devices) have the potential to aid behavior change through allowing individuals to engage with health-related content remotely, which may potentially address logistical challenges (eg, travel, work, and childcare) that arise when attending face-to-face interventions [11], which are barriers that young women in particular face [12]. A weight gain prevention program delivered via an app could also be scalable to UK family history clinics.

### Objectives

A search of the literature identified no evidence-based apps that covered weight gain prevention and all relevant health behaviors for women at increased risk of breast cancer [13]. Thus, with patient and public involvement from the target population, this research team developed a digital intervention including an app (the “health behavior intervention”) focused on preventing weight gain and improving health behaviors among women aged 18 to 35 years at increased risk of breast cancer. The protocol for this intervention has been described previously [13]. This intervention was tested in a feasibility study to assess the study procedures for a planned future efficacy study and test the acceptability and usability of the intervention over a 2-month period. An analysis of the use of the intervention is provided elsewhere [14].

A key component of feasibility is acceptability to the target audience [15]. This qualitative interview study explored the views of a subset of women on their experiences during and after taking part in the health behaviors intervention. The feedback from participants will inform the feasibility of effectiveness and cost-effectiveness research of the app-based intervention.

## Methods

The methods are reported in accordance with the Standards for Reporting Qualitative Research [16] (Multimedia Appendix 1) and the COREQ (Consolidated Criteria for Reporting Qualitative Research) guidelines [17] (Multimedia Appendix 2).

### Design

This research is nested within a single-arm intervention study (trial registration NCT05460650) [13]. Semistructured qualitative interviews asked participants for their experiences using the health behaviors intervention for a 2-month duration.

### Participants and Recruitment

Participants were female individuals who had taken part in the health behaviors intervention. Inclusion criteria for participation in the health behaviors intervention were female sex, age of 18 to 35 years, residence in the United Kingdom, a moderate or high risk of breast cancer [2], ability to communicate in English, and the ability to download and use an app. Participants were excluded if they had a previous diagnosis of breast cancer, a previous bilateral preventative mastectomy, or medical conditions that influence diet or weight. Further exclusion criteria for the trial are detailed elsewhere [13].

Women were recruited to take part in the health behaviors intervention via mailshot from the Manchester Family History Clinic and via social media posts to expand diversity within the recruited population and reach women outside of the Greater Manchester region. Prevent Breast Cancer (registered charity number 1109839) promoted the study via their social media feeds during the recruitment period. Following use of the health behaviors intervention for 2 months, a member of the research team (MP) contacted participants inviting them to take part in an interview about their experiences of taking part in the digital intervention. Women who had completed the study (n=35) [14] were purposively selected, aiming to recruit a range of ages, ethnicities, and levels of engagement with the app. Those participants who agreed to be interviewed gave their permission to be contacted by another member of the research team (REH) to arrange a suitable time for the interview. Of the 21 women who were approached for an interview, 1 (5%) later declined due to time constraints.

### Intervention

The health behaviors intervention consisted of a videoconferencing welcome event hosted on Microsoft Teams, an app with an embedded microsite containing educational topics, and a private Facebook group (Meta Platforms). Participants used the intervention for 2 months. The aim of the health behaviors intervention was to prevent weight gain (by encouraging behaviors such as healthy eating and physical activity) and improve other health behaviors associated with breast cancer, including reduced alcohol intake and smoking cessation.

A number of behavior change techniques were included in the app that have been shown to have efficacy in primary weight gain prevention settings. These techniques included self-monitoring of behaviors and frequent monitoring of body weight [18,19], goal setting [18,20], automated feedback on behaviors and outcomes [18,21], social rewards (ie, positive reinforcement included as part of the automated feedback) [21], and social support provided via the Facebook group [18,22]. The app interface was designed to allow users to record progress and see this in relation to their set goals. These behavior change techniques have all been found to be effective in weight gain prevention interventions in young adults [18] and in digital health interventions for young adults [21,22]. The health behaviors intervention is described in more detail elsewhere [13]. The immediate research team also had access to participants’ tracking data, and results of intervention use are described in a separate analysis [14]. Multimedia Appendix 3 includes screenshots of the app interface. The 3 intervention components are briefly summarized in the following paragraphs.

At the start of the study, participants were invited to attend one of several videoconferencing welcome events held on the Microsoft Teams digital meeting application, lasting up to an hour, with up to 10 other participants and a member of the research team (MP). The aim of these events was to (1) provide an overview of the study; (2) provide a simple outline of the evidence for the association among health behaviors, body weight, and breast cancer risk; (3) meet and build relationships with other participants involved in the study; and (4) build rapport with the research team.

The app included the functionalities for participants to log their weight, physical activity, diet, alcohol consumption, and smoking on a weekly, fortnightly, or monthly basis, and participants could set goals for themselves (Multimedia Appendix 3). The app also included an embedded microsite containing information on topics such as alcohol and its impact on breast cancer, the importance of fruit and vegetables in the diet, and how to limit weight gain during pregnancy. Participants could access the app beyond the 2-month study duration but were made aware that their use of the app after the end of the 2 months would not be monitored by the research team.

Alongside the app, participants were given the opportunity to join a private, hidden Facebook group to allow for access to social and peer support for behavior change and contact with the research team. Participants were encouraged to post within the group, and educational information on breast cancer and healthy behaviors was posted weekly by the research team to promote interaction. The research team acted as moderators and checked the group daily to respond to messages and comments.

### Procedures

One-to-one semistructured interviews were conducted by a female researcher (REH; research associate) who had a background in health psychology and training in qualitative methods and was independent from the study delivery team. REH described the aim of the research to participants as wanting to understand their experiences of being a part of the study and their feedback on the health behaviors intervention. Interviews took place on one occasion over the telephone between February 2023 and March 2023 within 1 month after participants had completed the study. There was nobody else present for the interview besides researcher REH and the participant. Full consent had already been obtained before the interview, although REH asked participants to confirm that they were happy for the interview to be audio recorded and transcribed before commencing the interview, and participants’ permission was audio recorded. Each interview was recorded using an encrypted audio-recording device, transcribed verbatim by a university-approved transcription company, and pseudonymized for analysis. The interviews lasted between 30 and 60 minutes. Transcripts were not returned to participants for comment. Recruitment was stopped when the researchers (REH, MP, DPF, and MH) felt that no new content was discussed (in what became the final 2 interviews) according to the concept of “information power” (ie, information from the sample that is relevant for the study [23]).

### Materials

A topic guide was used to organize the semistructured interviews, with open-ended questions and additional probes (Multimedia Appendix 4). Questions were asked in line with the overall study objectives [13]. These included participants’ experiences of taking part in the digital intervention, the usability of the app, the extent to which the intervention was useful in helping participants change their health behaviors, and the extent to which the intervention may have influenced participants’ health behaviors or feelings toward breast cancer. Although the topic guide was not pretested, the interviewer (REH) maintained an audit trail after each interview had taken place to document initial thoughts that may contribute to later analyses. Following the first 2 interviews, the interviewer (REH) amended the ordering of some of the interview questions to improve the flow (eg, asking about participants’ behavior changes at the end of the interview, after interviewees had discussed their suggestions for improvements to the app).

### Researcher Positioning

The interviewer and lead author (REH) was female, aged 29 years (and thus was familiar with the participants’ life stage and had life experiences in common with some participants), and had a BMI within the “healthy” range but, to the knowledge of the author, did not have a history of breast cancer in her family. She had worked in prevention research (including diabetes prevention and breast cancer prevention and early detection) for >5 years and had previous experience delivering behavior change interventions in the community, thus reflecting her positive views on disease prevention.

In total, 2 members of the research team who conceptualized this intervention were research dieticians specializing in health behavior change and breast cancer prevention (MP and MH), thus reflecting their positive views on weight gain prevention in this population, and both had >15 years of experience in this field. Other members of the research team were breast cancer clinicians working in breast cancer prevention and early detection (AH, DGE, and SJH) or had experience designing, developing, and evaluating apps and behavior change interventions in industry and academia (REH, DPF, AD, and JM). The backgrounds of the wider team may have influenced some of the questions asked in the interviews (eg, with more of a focus on the individuals rather than wider socioeconomic status constraints) and the interpretation of some of the findings.

### Analysis

Data were analyzed using inductive thematic analysis [24] and organized using the framework approach (Multimedia Appendix 5) to identify any differences in the findings across participants. As the researchers wanted to understand participants’ views and experiences of specific features of the intervention (including support, logging health behaviors, and educational topics) and their overall views of taking part in the intervention, data were analyzed from a critical realist perspective, which seeks to understand participants’ experiences but where participants’ interpretations define their subjective realities. Participants did not provide any feedback on the findings.

The data were analyzed by 1 researcher (REH) and discussed among the authors to identify themes relevant to the research questions, with illustrative extracts and interpretive themes refined through discussion. The NVivo software (version 12; QSR International) was used to facilitate coding and analysis of the data.

### Ethical Considerations

This study was reviewed and approved by the Wales Research Ethics Committee 3, Cardiff (reference 22/WA/0164). All participants provided full informed consent before they downloaded the app for the study. The wider study that this qualitative study is a part of is registered on the web at ClinicalTrials.gov (reference NCT05460650). There were no financial incentives for participating in the study. Interview data were pseudonymized at the point of transcription.

## Results

### Overview

The 20 interviewees comprised female individuals with a median age of 32 (IQR 27-35) years. The sample had little ethnic diversity but a good spread in terms of age range and engagement with the app (Table 1). The interviews lasted for a median of 37 (IQR 30-46) minutes.

**Table 1:** Participant characteristics (N=20).

Characteristic		Values
Age (y), median (range; IQR)		32.5 (23.7-35.6; 27-35)
**Ethnicity, n (%)**		
	White British	18 (90)
	Other	2 (10)
**English Index of Multiple Deprivation quintile, n (%)**		
	1 (most deprived)	1 (5)
	2	4 (20)
	3	7 (35)
	4	5 (25)
	5 (least deprived)	3 (15)
Baseline BMI^a^ (kg/m^2^), median (range)		24.2 (19.5-31.6)
Estimated lifetime risk of breast cancer (Tyrer-Cuzick model; %)^b^, median (range)		28.2 (17-50)
**Level of education, n (%)**		
	A-levels or post-16 qualifications	2 (10)
	Degree	6 (30)
	Postgraduate degree	12 (60)
**Recruitment method, n (%)**		
	Mailshot	14 (70)
	Instagram	6 (30)
**Attendance to Microsoft Teams welcome event, n (%)**		
	Yes	16 (80)
	No	4 (20)
**Member of private Facebook group, n (%)**		
	Yes	15 (75)
	No	5 (25)
**Engagement with the app, n (%)^c^**		
	High user	11 (55)
	Low user	9 (45)

^a^Underweight: <18.5 kg/m^2^; healthy weight: 18.5-24.9 kg/m^2^; overweight: 25-29.9 kg/m^2^; obesity: 30-39.9 kg/m^2^; severe obesity: ≥40 kg/m^2^.

^b^Near population risk of breast cancer: <17%; moderate risk of breast cancer: >17%-<30%; high risk of breast cancer: ≥30% [3].

^c^Participants were classified as “low” or “high” users based on the number of dates on which they entered information into the app. High users entered information on ≥6 dates, and low users entered information on ≤2 dates.

A total of 4 themes were generated from the analysis (*A “missed opportunity” in breast cancer prevention services*, *The pros and cons of being part of a community*, *The importance of an interactive app*, and *The different wants and needs of different age groups*). Quotes are presented with pseudonyms for the participants and their age.

### Theme 1: A “Missed Opportunity” in Breast Cancer Prevention Services

The women recounted their experiences of being told at a young age that they were at increased risk of breast cancer but then not having a service that they could access to help manage and reduce this risk until they were aged 35 or 40 years. Their accounts centered on feeling forgotten, “fobbed off” (Isabelle), and not always taken seriously by health care professionals. Stephanie described feeling like she was currently in “no man’s land,” where the knowledge of being at increased risk is at the back of her mind but there is little she can do until she can take risk-reducing medication when she reaches the age of 40 years:

You do feel a bit lost to be honest because, you know, you kind of get this letter from clinic and that’s it. You kind of are just sent on your way and then years tick by and I suppose you haven’t got that support in the interim.

Stephanie; aged 35 years

Many women recalled finding out that they were at increased risk of breast cancer at an early age, usually because of the diagnosis of a close family member. Some women reflected on this as a burden they had lived with for a long time, as Alice described: “...it feels like it’s leaning over you.” In contrast, Amelia described her breast cancer risk as a “future thing to worry about” as she did not want to ruminate about something that might or might not happen. Regardless of how the women perceived their own breast cancer risk, they were unanimous in that there was a gap in service provision for women of this age group, which was described by Isabelle, who found out she was at increased risk of breast cancer when she was aged 15 years, as a “missed opportunity.” Charlotte reported feeling let down by the National Health Service and the lack of funding or time that is invested in women’s health:

They can’t just kind of brush it off and say, oh, come in when you’re forty. That’s just not good enough to me.

Charlotte; aged 26 years

Therefore, the women described feeling dutiful to take part in the research study as it provided an opportunity to shape services for future women in a similar situation and prioritize research in women’s health:

I wanted to do something that might help other women and people in the future.

Olivia; aged 31 years

Therefore, taking part in this intervention provided some women with a sense of control and empowerment. It was an opportunity to learn more about how they could actively try to reduce their breast cancer risk and, thus, “taking matters into their own hands” (Zoe). Some participants described feeling reassured that they had not been completely forgotten about:

You feel a bit helpless, because it’s just a case of waiting and hoping that nothing happens in that time, which it could do. So yeah, I’d rather be doing something I guess, or exploring things that might help, rather than just sitting and waiting for a few years...So it seemed like this was something that I could proactively do, rather than just wait until I’m thirty-five.

Josie; aged 33 years

### Theme 2: The Pros and Cons of Being Part of a Community

The women had diverging views as to whether support from other women at increased risk of breast cancer was of interest to them. Naomi noted the lack of community available to women aged <40 years known to be at increased risk of breast cancer:

There’s a huge community for people who have had breast cancer, is there a community for people who are high risk but are going through the same thing?

Naomi; aged 26 years

Of those who were keen to connect with other women at increased risk of breast cancer, the Microsoft Teams welcome meeting was reported as the first opportunity to speak with other women in a similar position to them. The women described feeling reassured that there were others “in a similar boat” (Emily), which helped validate their own feelings when meeting like-minded women who could empathize with them:

I think it was more to just know. Like when I first started at the Welcome Teams meeting, it was just to know that there is other people in that situation because on a weekly basis I do worry and I check myself continuously and things like that. It’s kind of nice to know that there’s other people in that situation that are also of a similar age group.

Lucy; aged 24 years

Emily noted that support from other women with similar lived experiences has the potential to fulfill emotional support needs, which is notably different from the support she would receive from health care professionals at the family history clinic, although she perceived support from both sources as important:

I suppose [support from other women provides] some kind of context to your situation, so I think, you know, hearing other women who were about my age who’d got similar family history to me. You’ve kind of got what they experience and how they manage that kind of uncertainty, I thought that was quite useful, but wouldn’t necessarily get from a clinic appointment. So I think sometimes you miss the contact in a clinic appointment, and it’s all about reducing risk, which obviously is important but I also think that a bit of contact in how other women manage it, or live with it I guess, is useful.

Emily; aged 29 years

Most women who did attend the Microsoft Teams welcome meeting expressed that they would have liked to stay in touch with the women on the call via another follow-up meeting. They suggested that the 5 minutes that they had to talk to each other within the meeting could be increased in future group calls to enable them to establish more of a rapport with the other women.

In contrast, others viewed a support system or community as potentially unhelpful. Josie recalled that speaking with others on the Microsoft Teams call brought to her attention that others had started a family before the age of 30 years, but Josie was unaware that this could contribute to breast cancer risk reduction and had not yet had children:

...so in some ways it was good, because it was nice that people were there who’d had similar experiences of obviously having had breast cancer in their family and stuff. But in other ways it kind of brought home some of the things that people have done to reduce the risks that I hadn’t.

Josie; aged 33 years

Conversely, others presented fatalistic views and perceived breast cancer as inevitable, thus viewing support as redundant “because although support is great, no one can change your outcome” (Charlotte). Alice explained that she would not view interacting with other women as “support” until she was diagnosed with cancer:

But I think to have it [support] there prior to that [diagnosis] and then having the knowledge of these people that you might have built a relationship around you getting cancer and you might just feel like you’re on a waiting list to get cancer. Do you know what I mean?

Alice; aged 35 years

Therefore, it was suggested that the opportunity to meet others on the web via Microsoft Teams could be optional; some might want an introduction to the study without sharing their own experiences or hearing others’ stories. Even those who expressed a desire to connect with other women acknowledged that support will look different for different people and not everyone will want this:

I don’t want to get into a hole about it. I’ve said that I understand my family history, I understand, you know, it’s a possibility it could happen to me. I think being told about it, or being ah, kind of reminded about it often, it didn’t really appeal to me. And I think for my sake and my mental health, I just find it easier knowing what I know and not being kind of reminded of it constantly.

Carly; aged 28 years

### Theme 3: The Importance of an Interactive App

Should such an intervention be rolled out, all the women discussed the need for an interactive app that provided trustworthy information specific to breast cancer risk reduction. Although most of the women described themselves as quite knowledgeable about breast cancer risk factors, many recalled something that they had learned and implemented as a result of engaging with the intervention. For example, in the following quote, Rebecca recalls learning about the link between breast cancer and alcohol consumption, which prompted her to reflect on her own alcohol intake:

It just kind of made me think a little bit more about my own alcohol intake and any changes that I can make to my own intake of it really because I was surprised about how, like, I think it was the amounts of units that women were supposed to drink in a week or something kind of surprised me at how sort of low it was in comparison to my own intake.

Rebecca; aged 23 years

However, others mentioned wanting more in-depth information about breast cancer prevention and risk management in addition to health behaviors, including information about how the contraceptive pill influences breast cancer risk and how to perform a breast check:

So, like you know, like self-breast examination and what are the steps you can take to, you know, if you find a lump, if you find swelling so where to go and like that.

Amara; aged 35 years

The desire for more interactive app features was described as another way to increase engagement with the intervention. This ranged from including more tailored information relevant to yearly events to the functionality to link to other apps:

If the notifications pulled that through that would be great. And even if they engage in stuff wider about evidence based things, about national drives that are happening, or like breast cancer awareness week, something like that.

Stephanie; aged 35 years

So I like apps that integrate everything. So, I’ve got a fitness tracker. So everything that I do like if I record a run, it pings on about three different apps. So like if it did that. If it linked with fitness devices, if it linked with other health apps, so that all the information fed into one place. So potentially maybe you wouldn’t have to re-input it, if it just pulled through other apps, if that makes sense.

Grace; aged 34 years

During the study, members of the research team posted educational topics on the Facebook group each week. For some, this was a useful prompt to engage with content that was often missed on the app. Others who were not part of the Facebook group had fewer recollections of the educational content and, therefore, suggested that the app itself could include notifications of the weekly educational topics alongside quizzes and challenges to facilitate learning. The women viewed the ideal app as including tracking, education, and support features in one place to increase interactivity:

I think on the app if things were sort of revealed weekly, that would be good. Rather than just having all the information there together. Just because like I can get busy, you know, get sidetracked but if it’s like a weekly thing...I think that can help with the engagement. Help keep you looking for if there’s something new coming up, rather than this just sort of static app that, you know, you very easily could just sort of get to put to the side. I think people need reminding.

Holly; aged 35 years

I think for me as well almost having like an interactive element would be really helpful, so, you know, it’s almost like testing your skills but like a quiz or something. And just to make it as I say a bit more interactive, so you feel like you’re, you know, you’re taking the learning but then you’re also embedding it a little bit as well.

Amelia; aged 32 years

Most of the women expressed the need for an intervention such as this one to include more tailored signposting to services available in their local area (eg, running groups and weight loss groups) pointing them to the practical support available to maintain a healthy lifestyle. Thus, it would be up to the individuals themselves whether they wanted to seek further information and support for their health behaviors at a time when they might need them.

### Theme 4: The Different Wants and Needs of Different Age Groups

Participants felt that, if an intervention such as this one was to be rolled out, it would need to be appropriate for the different age groups between 18 and 35 years engaging with the family history clinic, who each might have different wants and needs from such an intervention “because what I might want might be completely different to what a twenty-two-year-old these days want” (Alice; aged 35 years). Thus, when developing a digital intervention for women aged 18 to 35 years, aspects of the intervention would need to be modified for different age ranges within this group of women. For example, raising awareness about the modifiable risk factors was perceived by the women to be important for all age groups, whereas Rebecca described that tracking health behaviors on an app might not always appeal to younger age groups:

As I’m only twenty-three and I was told that I wouldn’t really be eligible for any sort of monitoring or anything until I was like in my thirties, for me, it seems like something that is so far off, it’s hard for me to feel any, like any kind of reasoning to do it [tracking health behaviours] right now. ... I’d be much more inclined to read the information. Because I think, you know, that’s relevant at any stage of life and it’s interesting as well, to kind of know things. So I’d be happy to read more about that and get the information and stuff. But the logging for me, it’s just not personally, something I’m interested in doing right now.

Rebecca; aged 23 years

Therefore, the women’s accounts continually emphasized that there is not a “one size fits all” approach and such an app would need to be customizable to provide the women with the autonomy to manage their health at different stages of their lives, echoing accounts from the previous themes. The women gave examples of having options to receive notifications or reminders from the app, seek further information on topics that were relevant, meet with other women at increased risk of breast cancer, receive signposting for health behaviors, or track different health behaviors on the app. This highlights the complexity of needs that such an app would need to accommodate. For example, Sophie reflected on the importance for women to choose what kinds of app notifications they want to receive:

I think that it could be optional for the user to decide whether they want the information. And then it’s like if they change their mind, they’ve got the option to stop those notifications, so that it suits the person using it.

Sophie; aged 25 years

The women also discussed the need for wider education about health behaviors and breast cancer risk. Amber reflected on the current limited education on breast cancer risk reduction and the need for increased societal efforts to raise awareness:

I think there’s a lot of people out there that don’t realise how much their lifestyle can impact their breast cancer risk. Obviously there’s many, many risks that cannot be changed by an individual, you know, their genetics, etc. However, some people don’t realise that they are affecting their risk by their health behaviours.

Amber; aged 29 years

Thus, in addition to an interactive app, social media was regarded as another appropriate platform to raise this awareness. Zoe stated that choosing to engage with a social media app is more “automatic” compared to an app to track health behaviors, suggesting that bite-sized information via videos might be more beneficial for younger age groups who are higher social media users:

Well, they’re on it all the time, aren’t they [laughs]? So if you’re on it and it’s popping up and if you look at one thing, something pops up so I don’t know. Yeah, maybe little reminders on there like little posts, you know, remember to check yourself, remember smoking increases risks of X, Y and Z, you know. Keep your BMI healthy. Try and, you know, try and do a bit of exercise. Do you know like those kind of little posts?

Zoe; aged 35 years

This was perceived as important for all women regardless of their family history:

Because, breast cancer is an issue, not just for people who it’s running in the family, it’s a huge issue across the board.

Grace; aged 34 years

Cervical screening was one avenue that the women felt could help raise awareness of health behaviors and breast cancer in younger women given that women are invited to attend cervical screening from the age of 25 years. The women also suggested that nonclinical settings such as gyms and national campaigns should be raising awareness about the importance of health behaviors:

But yeah, I think that’s the key, there’s always going to be people who are seeking it out and interested in, you know, or a bit more aware, but there’s also people who sort of at similar risk, like in the same group as us, who perhaps will be a bit more reluctant to sort of address their behaviours and things. So I think just getting it out there in a more sort of—in a less clinical setting perhaps, I think that’s a big thing.

Isabelle; aged 34 years

## Discussion

### Principal Findings

The accounts of the women in this study drew on the lack of services available for women aged 18 to 35 years and the lack of education and awareness raising in breast cancer risk reduction for younger women, thus highlighting such an intervention as a missed opportunity in breast cancer prevention services. The women had diverging views regarding the extent to which they would value being part of a community with other women at increased risk of breast cancer. If an intervention were to be rolled out, the women described various interactive features that would promote engagement (eg, quizzes, unlocking educational content over time, and app notifications). However, the women also described the complexity of such an intervention aimed at multiple age groups with different wants and needs. Thus, a customizable app with options to engage with different intervention features to varying degrees (eg, education, tracking, and support) was perceived as vital to allow women the autonomy to manage their health at different stages of their lives.

### Comparison With Previous Literature

There are many qualitative studies on weight loss and weight loss maintenance [25]; however, to date, very few qualitative studies have focused on primary weight gain prevention in women at increased risk of breast cancer. Previous research with women aged 26 to 35 years at increased risk of breast cancer explored women’s ideas about a hypothetical weight gain prevention intervention to reduce breast cancer risk, which was welcomed [26]. This study builds on those findings and presents reactions to a digital intervention targeting weight gain prevention and modifiable health behaviors for women at increased risk of breast cancer; the women in this study perceived this not only as acceptable but also as an important intervention that is missing from current service provision.

Other qualitative research among women at high risk of breast cancer has suggested that some see little value in making changes to their health behaviors if they are well [27], and a recent systematic review found that many women at high risk of breast cancer often view it as “inevitable,” including some misunderstandings of their own breast cancer risk factors [28]. Further qualitative research has found that women are sometimes uncertain of the preventative value that positive health behaviors can have, particularly when they have a family history of breast cancer [29]. However, this study found that, when the women learned about the links among weight reduction, health behaviors, and breast cancer risk, they reported that they had started implementing behavior changes that allowed them to gain a sense of control over their risk, in line with the study by Wright et al [30].

Previous qualitative research has highlighted the importance of providing women with credible information about weight control and health behaviors in relation to breast cancer risk [26,30]. Recent qualitative research has also reported that providing advice on health behaviors was perceived by some women aged 47 to 74 years as an acceptable means of controlling breast cancer risk [31]. This study found that the proposed digital intervention was also perceived as an acceptable way to provide credible information for younger women. However, the women in this study also emphasized the importance of an interactive app, such as quizzes and push notifications, to increase the usefulness of the intervention. This is in line with user engagement research in the wider digital behavior change literature, which has reported that sending push notifications containing tailored health messages was associated with greater engagement with a mobile health app [32].

### Implications

The women in this study found the health behaviors intervention acceptable as a cancer risk reduction strategy, and an app was considered an acceptable format of delivery. However, such an intervention will need to be customizable to the different wants and needs of women at different stages of their lives. Subject to future feasibility studies, a digital health intervention for women at increased risk of breast cancer could be a scalable behavior change strategy for all UK family history clinics. Further research would need to assess whether this leads to additional supportive needs and resource requirements for participating clinics, as well as the skill set of staff and the capabilities required to meet this need if this intervention is part of a sustained future service. Current initiatives and research in breast cancer risk assessment are seeking to identify more young women at increased risk [33]. For example, there is now a greater awareness that family history clinics are not capturing women at high risk of breast cancer who have no known family history of breast cancer [34], and there have recently been calls for primary care involvement in identifying these target populations [35]. Therefore, it is likely that the number of women aged <40 years attending UK family history clinics—and, hence, the demand for such an intervention—will increase.

There is potential for further development of this intervention. The women in this study particularly valued information about alcohol consumption and breast cancer risk as many reported being previously unaware of this link. Clinicians should explain how women can modify specific health behaviors, including alcohol consumption, and why this is important for modifying future breast cancer risk. In addition to health behaviors, the women also reported wanting more information about other breast cancer risk factors such as the contraceptive pill, risk-reducing medications, and breast self-examination. Furthermore, including SMS text messaging with novel information that requires less active seeking of information and increasing the interconnectivity of the app with existing technologies are other fruitful avenues to explore for future developments. Strengthening the social media element of the intervention may help increase engagement for some groups of women. Given the merit that the women attributed to social media in raising awareness about health behaviors and breast cancer risk, research could also evaluate young women’s engagement with educational posts about breast cancer risk on social media. This might establish whether social media can fill a gap in awareness raising in those who might not seek out information or regularly engage with an app.

This research also highlighted the lack of support or community in place for young women known to be at increased risk of breast cancer. Although the women had differing views as to whether such a community was of interest to them, it was acknowledged that preferences for support could change over time depending on personal circumstances and life stage, thus highlighting a clear unmet need for this population. Therefore, future developments of this intervention should consider how to incorporate an optional community for those who would benefit, for example, through group meetups or campaigns.

Future evaluative research with the app should embed qualitative work to understand the barriers to engagement experienced by women of a low socioeconomic status and from ethnic minority groups; these groups are disproportionately affected by cancer but are underrepresented in cancer prevention research [36]. Future research could also consider whether this intervention is applicable to other groups, such as women at increased risk of breast cancer aged >35 years or people with Lynch syndrome.

### Strengths and Limitations

This analysis involved researchers from diverse backgrounds, and the lead author was independent from the previous app development [13] and from the study team, thereby reducing the likelihood of the results being influenced by the wider team. There was a fairly even spread of level app engagement across interviewees, enabling a range of views to be captured on reasons for engagement or lack thereof. The interviews were conducted in a timely manner at the end of the intervention, within 1 month of the women completing the study.

We acknowledge the limitations of this study. Although efforts were made to secure a broad representation of participants regarding age, ethnic groups, and engagement with the intervention, the sample had little ethnic diversity, and we were unable to interview women who did not opt to join the study. The sample of interviewees was highly educated; the women described themselves as fairly knowledgeable about their health behaviors, and some were already implementing such behaviors at the time of taking part in this intervention. Therefore, the women in this study were likely to be a motivated sample, and caution should be taken when transferring these findings to the wider population of women at increased risk of breast cancer. The interviewees discussed their experience of 2 months’ participation in the intervention. We acknowledge that this pragmatic acceptability study was short and that future research will need to assess longer-term interventions to assess the efficacy of the app for sustained behavior change.

### Conclusions

There is a gap in prevention services for young women aged 18 to 35 years at increased risk of breast cancer. The women perceived a digital intervention incorporating education, tracking, and support to be an acceptable way to manage health behaviors and weight gain prevention for this target population. However, such an intervention would need to be customizable to meet the wants and needs of different age groups of women aged between 18 and 35 years engaging with family history clinics at different life stages during this period.

## Appendix

Multimedia Appendix 1 Standards for Reporting Qualitative Research checklist.

Multimedia Appendix 2 COREQ (Consolidated Criteria for Reporting Qualitative Research) checklist.

Multimedia Appendix 3 Screenshots of the app interface.

Multimedia Appendix 4 Interview topic guide.

Multimedia Appendix 5 Coding framework.

## Abbreviations

COREQ: Consolidated Criteria for Reporting Qualitative Research

## Data Availability

The data sets generated during and analyzed during this study are available in the University of Manchester Figshare repository [37].
