# Supplementary material for: Experiences of a Digital Behavior Change Intervention to Prevent Weight Gain and Promote Risk-Reducing Health Behaviors for Women Aged 18 to 35 Years at Increased Risk of Breast Cancer: Qualitative Interview Study
Source: JMIR Cancer. 2024 Nov 25;10:e57964. doi: 10.2196/57964 (PMC11629029; doi:10.2196/57964)
Supplement: Multimedia Appendix 3 [file cancer_v10i1e57964_app3.docx]

**Multimedia Appendix 3: Screenshots of App Interface**

**Home page**

**
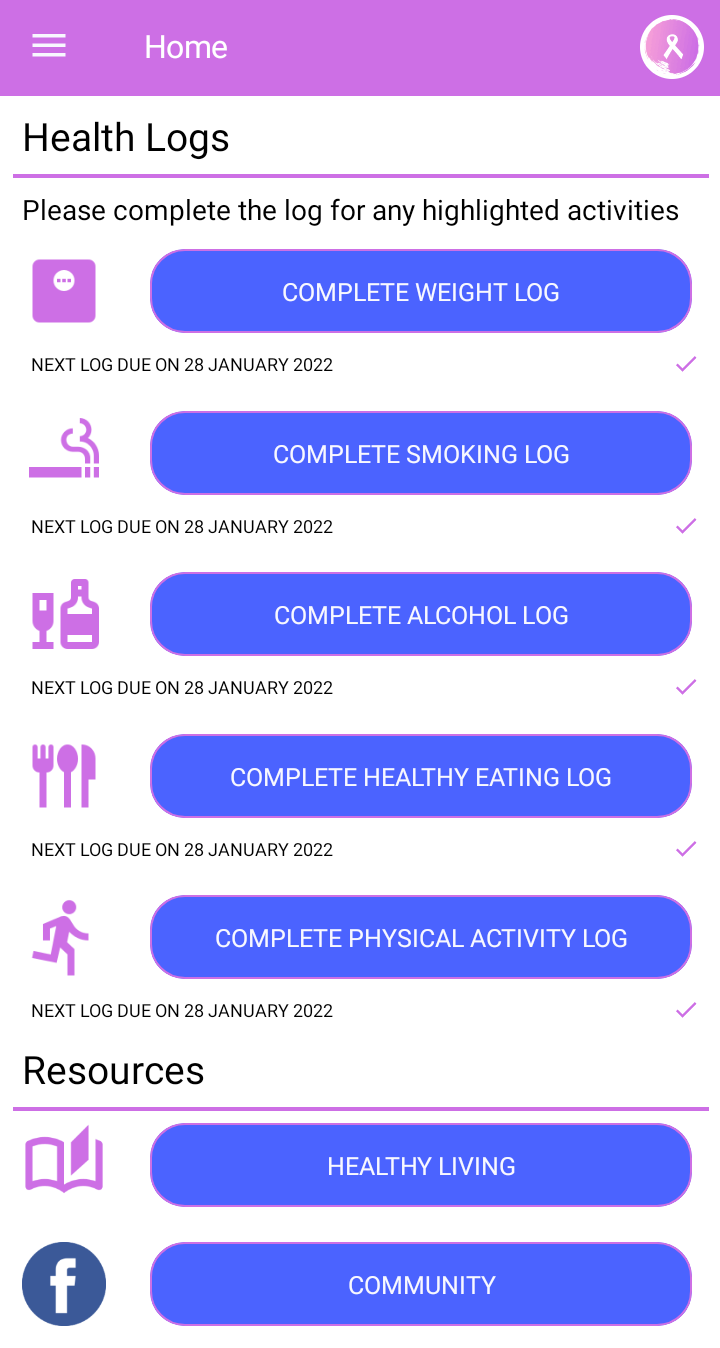
**

**[Note: Access to the microsite educational content is accessed via the ‘Healthy Living’ tab in the Resources section of the home page].**

**Enable health logs**

**
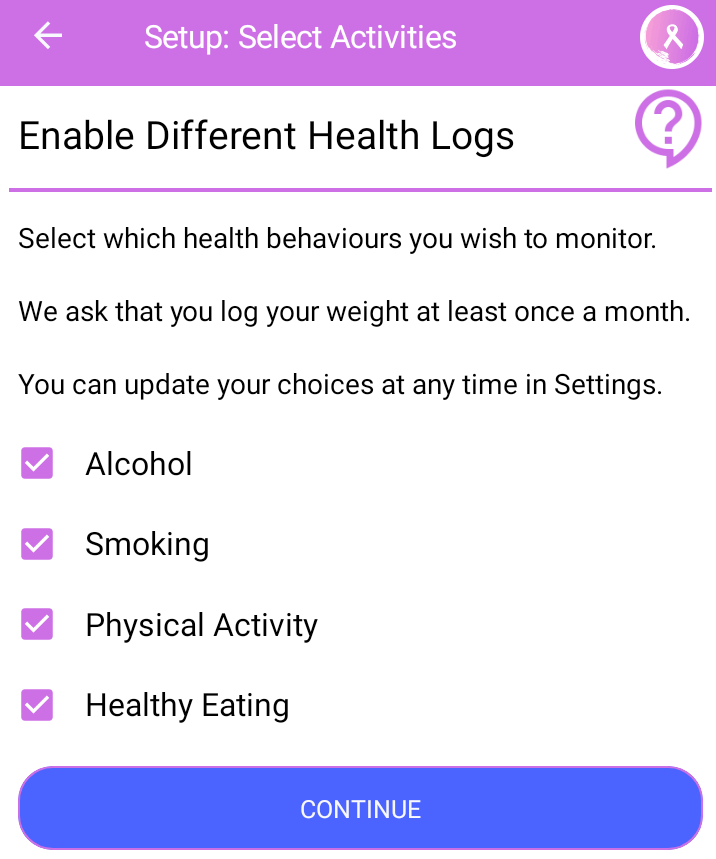
**

**Health log schedule**

**
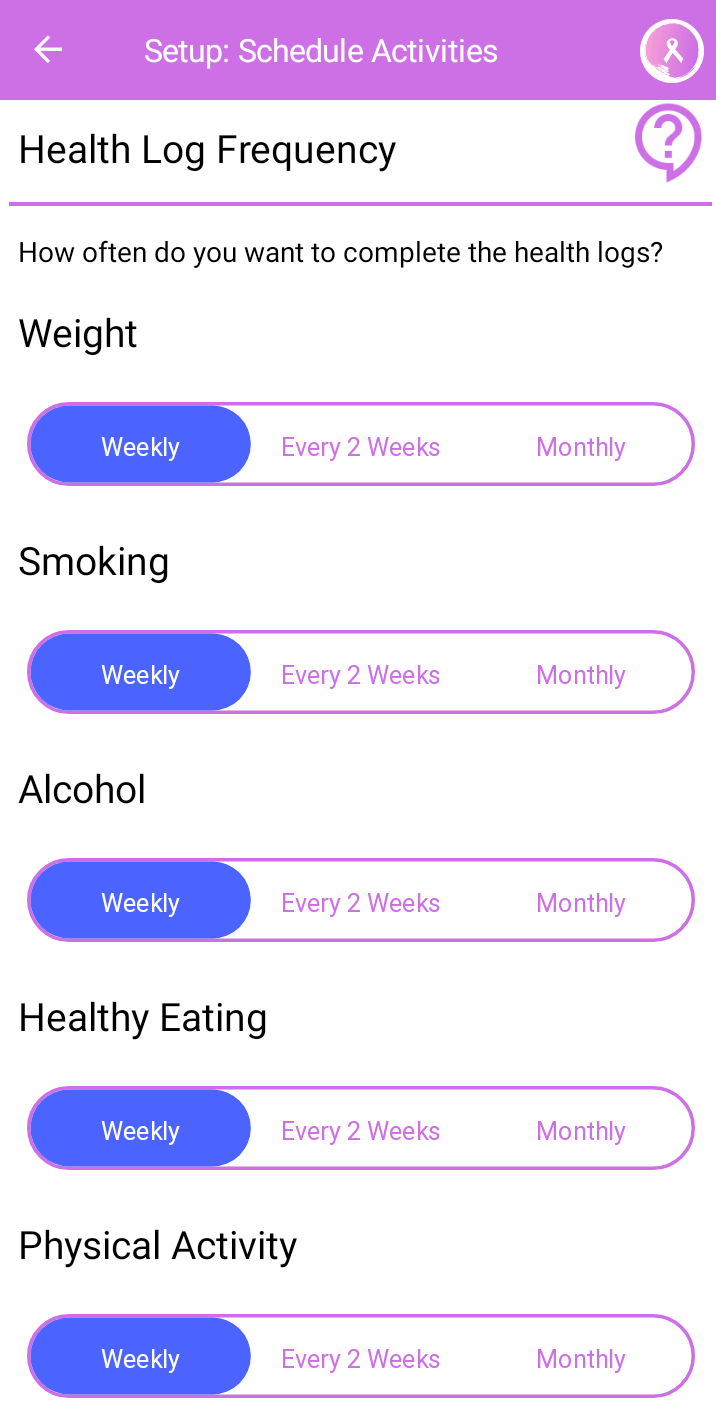
**

**Setting goals**

**
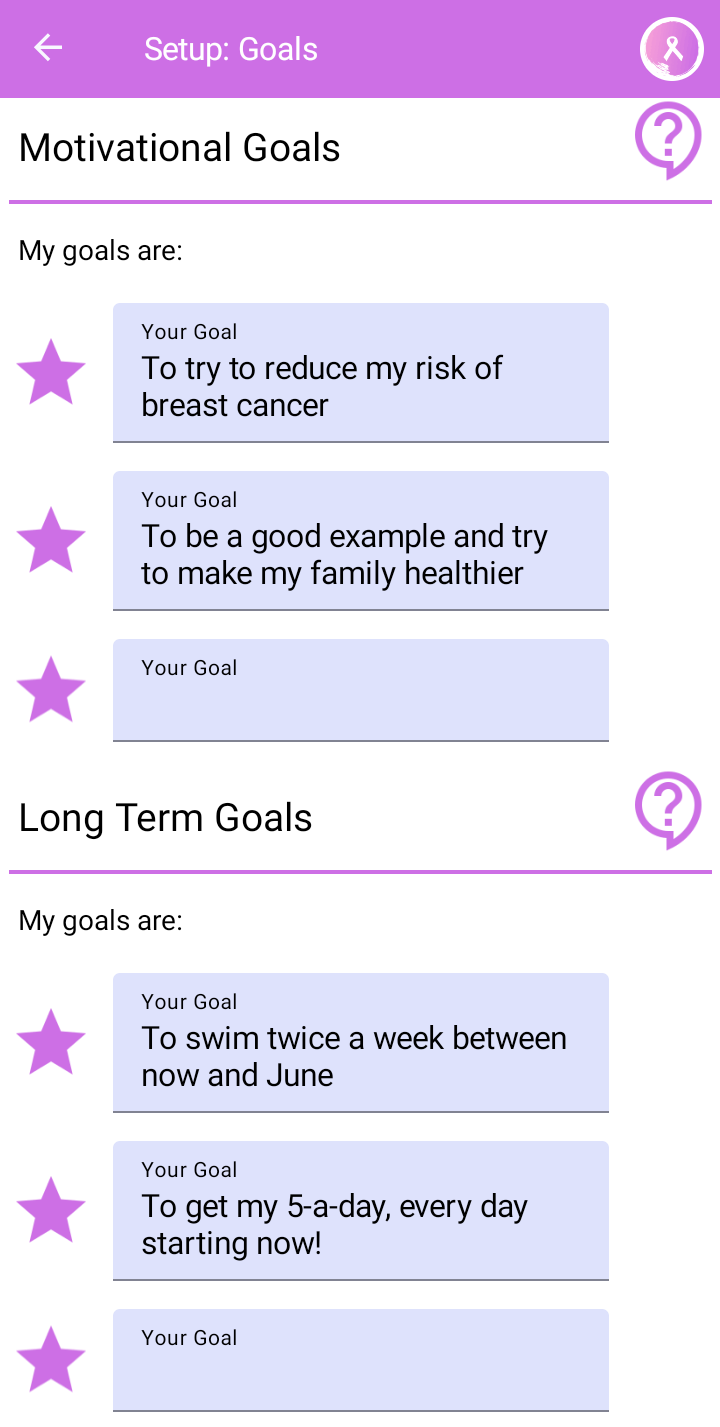
**

**
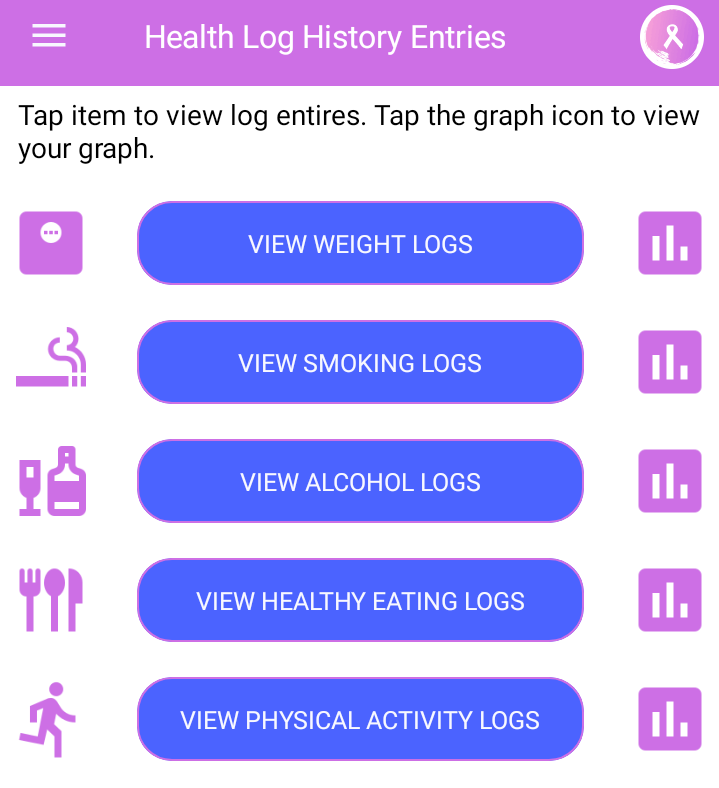

Health log history**
