# Supplementary material for: Experiences of a Digital Behavior Change Intervention to Prevent Weight Gain and Promote Risk-Reducing Health Behaviors for Women Aged 18 to 35 Years at Increased Risk of Breast Cancer: Qualitative Interview Study
Source: JMIR Cancer. 2024 Nov 25;10:e57964. doi: 10.2196/57964 (PMC11629029; doi:10.2196/57964)
Supplement: Multimedia Appendix 4 [file cancer_v10i1e57964_app4.docx]

**Multimedia Appendix 4: Interview Guide**

Introduction – consent and opportunity to ask questions

- Thank participant for time & introduce self.
- We are asking for the views of people who have been using the Healthy Behaviours App. We expect the interviews will last no longer than an hour.
- If at any time during the interview you do not wish to answer a question that’s okay.
- I would like to record our conversation. The recording will be typed out, but everything you say will be anonymous.
- If, at any stage, you wish to stop or pause the audio recorder, please let me know.
- Do you have any questions at this stage?

*Turn on recorder: Can I ask you to please confirm that you are happy to take part in the interview and that you are happy for me to record our conversation?*

Study recruitment

1. How did you hear about the study? Via invite from the Family History Clinic in the post or online?
2. What interested you to take part in the study?
   1. Can you tell me if there were any other influences on you taking part in the study? E.g. family member, FHRPC staff, etc.

Usability/acceptability of app

1. How did you find downloading the app and the registration process?
   1. What was easy/difficult during registration?
   2. Did you need any support in getting the app set up?
2. Did you take part in the Microsoft Teams starter group session?
   1. If so, why? What were your thoughts on the session?
   2. If not, why not?
   3. [If attended] is there anything about the session that you feel would be useful to change?
3. What parts of the app were the most useful for you?
   1. Why?
   2. Is there anything else that you found useful? Why?

*Then prompt if needed*:

- Logging weight?
- Logging behaviours? (physical activity, healthy eating, alcohol, smoking)
- Healthy living education resources?
- Downloadable resources?
- Videos?
- Facebook community?

1. What parts of the app are least useful for you/didn’t like?
2. How did you find the educational content e.g., weekly topics, videos?
   - 1. Can you recall any educational topics that you found particularly useful?
3. Did you use the Facebook support group? If so, why/how important was this to you? If not, why not?
   - 1. What could we have done to run this group better?
     2. Do you have any suggestions for what support could be offered for this intervention going forward?

Behaviour change

1. What have you learnt about breast cancer and health behaviours since you have started using the app?
   1. [Optional] How credible did you find the information on the app?
2. Looking back over the last two months, has the way you think about your risk of breast cancer risk changed? Why/why not?
3. Have you changed any health behaviours since you started using the app?
   1. What behaviours? Why?

Motivation to use the app long-term

1. [If they say they might get some value from using this app long-term]:
   1. What would encourage you to continue using the app?
   2. What new features might encourage you to use this more?

*Prompts if needed:*

How would you feel about linking the app to other wearables? (e.g. Fitbit, Apple Health)

How would you feel about being able to compare your weight and other health log results to those of other women using the app?

- How would you feel about receiving feedback on how changes to your health behaviours are changing your personal risk of breast cancer and other diseases?
- [FHRPC participants only] How would you feel about the Family History Clinic clinicians (doctors and nurses that you may have met in clinic) looking at your health log data?

How do you feel about being able to contact a health care professional from the Family History Clinic via the app?

How to improve the app

1. What features might be helpful for future versions that would have helped you engage more or get more out of this app?
   1. What might other people get from this app?
2. How easy was the app to use, when compared with other apps on your phone?
   - 1. How easy was the process of inputting your PA/diet/other information into the app?
     2. How frequently did you choose to log your health and why?
        1. Has anything got in the way of you logging at the frequency you chose to start with?
        2. What do you feel comfortable logging? What don’t you feel comfortable logging? What would make you feel more comfortable logging?
3. Did you experience any issues with the app (i.e., working slowly, crashing)? If yes, what did you experience?

Final comments

1. Is there anything we haven't talked about that you'd like to mention?
