# Supplementary material for: Experiences of a Digital Behavior Change Intervention to Prevent Weight Gain and Promote Risk-Reducing Health Behaviors for Women Aged 18 to 35 Years at Increased Risk of Breast Cancer: Qualitative Interview Study
Source: JMIR Cancer. 2024 Nov 25;10:e57964. doi: 10.2196/57964 (PMC11629029; doi:10.2196/57964)
Supplement: Multimedia Appendix 5 [file cancer_v10i1e57964_app5.docx]

**Multimedia Appendix 5: Coding Framework**

**Aim:** To explore participants’ experiences of taking part in the health behaviours intervention.

Framework was split into the following categories: ‘General experiences and study registration’, ‘Support’, ‘Logging behaviours and weight’, ‘Educational content’ and ‘Experiences of app’ to explore participants’ views and experiences of specific features of the intervention.

**GENERAL EXPERIENCES AND STUDY REGISTRATION**

| **Code *(description)*** | | | | | |
| --- | --- | --- | --- | --- | --- |
| Gap in service provision  *Lack of available support/ services for women aged 18-35 years at high risk of breast cancer* | Perceptions on risk of breast cancer  *Participant’s general views on how ‘controllable’ breast cancer risk is* | Family members with breast cancer  *Info/ context on participants’ family history of breast cancer* | More awareness of breast cancer risk factors needed  *Participant’s general views on breast cancer prevention awareness* | Willingness to support research  *Participants expressing an interest to contribute to research in breast cancer prevention to help others* | Registration  *How participants found out about the study, experiences with recruitment, registration and consent procedures* |
| **Sub-codes** | | | | | |
| Taking control of personal risk | Busy lives – lack of time | Burden of breast cancer risk | Suggestions to increase general awareness |  |  |

**SUPPORT**

| **Code *(description)*** | | | | | |
| --- | --- | --- | --- | --- | --- |
| Microsoft Teams event  *Participant’s views on the Teams event at the start of the study* | Facebook group  *Participant’s views on the running/ engagement/ interaction/ management of the Facebook group* | Advantages of support  *Participants’ general views on the advantages of providing support/ community for young women at risk of breast cancer* | Disadvantages of support  *Participants’ general views on the disadvantages of providing support/ community for young women at risk of breast cancer* | Support might look different for different people  *Views expressed by participants on the different types of support people may need at different life stages* | Suggestions for more support  *Participants’ suggestions for other forms of support/ additional support that could be offered in the intervention* |
| **Sub-codes** | | | | | |
| Advantages of Teams support (e.g., overview of study, meeting other women)  Disadvantages of Teams support (e.g., group size, patient confidentiality) | Facebook group a prompt for educational content  Interaction with Facebook group |  |  | Not everyone wants support  Different needs for different generations |  |

**LOGGING BEHAVIOURS AND WEIGHT**

| **Code *(description)*** | | | | |
| --- | --- | --- | --- | --- |
| Advantages of logging  *Participants’ general views on the advantages of logging health behaviours in the app* | Disadvantages of logging  *Participants’ general views on the disadvantages of logging health behaviours in the app* | Logging food  *Participants’ experiences of logging food/ diet in the app* | Logging physical activity  *Participants’ experiences of logging physical activity in the app* | Logging weight  *Participants’ experiences of logging weight in the app* |
| **Sub-codes** | | | | |
|  | Suggested improvements for logging in the app |  |  |  |

**EDUCATIONAL CONTENT**

| **Code *(description)*** | | | | | |
| --- | --- | --- | --- | --- | --- |
| Engagement with educational content  *Participants’ descriptions of how they engaged with the educational content* | Positive views of educational content  *Participants’ positive views and experiences of reading/ engaging with the educational content* | Negative views of educational content  *Participants’ negative views and experiences of reading/ engaging with the educational content* | More educational content requested  *Suggestions for other educational topics that would be useful to include* | Educational content – recall of what was learned  *Participants’ recall of what they learned when engaging with educational content* | Behaviour changes reported  *Specific changes participants reported to make to their behaviours as a result of engaging with the intervention* |
| **Sub-codes** | | | | | |
|  |  |  |  | Already aware of breast cancer risk factors | Already implemented behaviour changes prior to study |

**EXPERIENCE OF APP**

| **Code *(description)*** | | | |
| --- | --- | --- | --- |
| Positive experiences of the app  *Participants’ positive views and experiences of using and interacting with the app* | Negative experiences of the app  *Participants’ negative views and experiences of using and interacting with the app, including any technical issues experienced* | Suggested features that would encourage long-term use  *Participants’ suggestions for how the app could be improved, and features that would increase engagement and user experience* | Use of other technology  *Participants’ usage of other apps to keep track of their health and comparisons with these other apps* |
| **Sub-codes** | | | |
| Intervention has provided the ‘why’ to focus on health | No notifications  App is very basic |  |  |
